# How exercise frequency affects BMI: a nationwide cross-sectional study exploring key influencing factors, including dietary behavior

**DOI:** 10.3389/fpubh.2024.1514805

**Published:** 2025-01-15

**Authors:** Ming Zhang, Qinyi Guan, Jianrong Mai, Si Li, Chengwu Liu, Ling Zhou, Lina Lin, Kaisheng Teng

**Affiliations:** ^1^Department of Physical Education, Guangzhou Xinhua University, Dongguan, China; ^2^School of Public Health, Guangxi Medical University, Nanning, China; ^3^Department of Pathology, School of Basic Medical Sciences, Guangzhou Health Science College, Guangzhou, China; ^4^Department of Cardiothoracic Surgical ICU, The First Affiliated Hospital of Sun Yat-sen University, Guangzhou, China; ^5^School of Public Health and Health Professions, Guangzhou Health Science College, Guangzhou, China; ^6^School of Nursing, Guangzhou Xinhua University, Guangzhou, China

**Keywords:** body mass index, exercise, dietary behavior, mediation effect, influencing factor

## Abstract

**Purpose:**

Body Mass Index (BMI) is an important indicator for assessing obesity and related health risks. With the rapid socio-economic development and changes in lifestyle, abnormal BMI (such as underweight, overweight, and obesity) has become an increasingly serious public health issue. This study aims to explore the impact of exercise frequency on BMI among Chinese adults aged 19 to 59, and to analyze the role of dietary behaviors in regulating BMI, providing a basis for BMI intervention strategies.

**Method:**

The study employs a multi-stage sampling method across 23 provinces, provincial capitals, and four municipalities in China, randomly selecting 120 cities from each region. Online surveys were conducted using Wenjuanxing by trained surveyors.

**Result:**

A total of 8,611 individuals participated in the survey. Among them, 1,066 (12.38%) had a BMI < 18.5, 5,354 (62.18%) had a BMI between 18.5 and 23.9, and 2,191 (25.44%) had a BMI ≥ 24. Factors such as gender, age, marital status, monthly household income, smoking, and alcohol consumption significantly affected BMI (*p* < 0.05). The overall impact of exercise on abnormal BMI was −0.003, with a direct effect of −0.005. The mediating effect of dietary behaviors between exercise and abnormal BMI was 0.002, accounting for 92.48% of the total effect.

**Conclusion:**

This study highlights the widespread prevalence of abnormal BMI among individuals aged 19 to 59 in China. A single exercise intervention may be insufficient to effectively improve abnormal BMI; thus, it should be combined with strategies aimed at enhancing dietary behaviors.

## 1 Introduction

Body mass index (BMI) is calculated by measuring an individual’s weight (kg) and height, then applying the formula weight^2^/height^2^ (kg/m^2^) (1). Although BMI does not comprehensively reflect body composition, distinguish between fat and muscle, or account for differences in fat distribution, it is still widely used in clinical practice and research due to its simplicity, low cost, and high convenience. The Chinese Obesity Working Group recommends defining a BMI range of 18.5–23.9 kg/m^2^ as normal, while categorizing other BMI levels as abnormal (2). Within the abnormal BMI range, a BMI ≥ 24.0 kg/m^2^ is classified as overweight, a BMI ≥ 28.0 kg/m^2^ as obese, and a BMI < 18.5 kg/m^2^ as underweight^2^. A BMI < 18.5 kg/m^2^ and BMI ≥ 24 kg/m^2^ represent two extremes of health status: undernutrition and overweight, both of which have adverse effects on health (3). Over the past four decades, the number of obese adults has more than quadrupled, and the number of overweight individuals has also more than doubled (4). However, there is currently limited research on a BMI < 18.5 kg/m^2^. In fact, a BMI < 18.5 kg/m^2^ is more common in Asia than in Western countries, and low BMI is frequently associated with health issues such as malnutrition, chronic diseases, and compromised immune function (5, 6). Abnormal BMI adversely affects individual health and imposes a substantial medical and economic burden on society (7–9). However, the global prevalence of abnormal BMI is rising annually, underscoring the importance of focusing on abnormal BMI for public health (1, 10, 11). In addition, BMI is influenced by factors such as sex, age, and ethnicity (12–14). Given the vast size and diversity of the population, the investigation of the distribution and characteristics of abnormal BMI among Chinese residents is of significant public health importance.

With the rapid development of China’s economy and the progression of urbanization, significant changes have occurred in residents’ exercise habits and dietary behaviors. These changes have had a substantial impact on BMI levels. Exercise refers to physical activities aimed at enhancing physical health and is closely associated with an individual’s BMI level (15). A cohort study involving 5,159 Chinese residents aged 50 and older found that physical activity is significantly associated with the attenuation of long-term changes in BMI. This suggests that engaging in physical activity may aid older adults in maintaining healthy weight regulation (16). Another study, comprising 84 randomized controlled trials (RCTs) with 4,836 participants, found that regular exercise significantly reduces visceral fat in overweight and obese individuals (17). Dietary behaviors are another factor influencing an individual’s BMI level, characterized by consistent patterns and habits in eating. A study involving 4,249 residents aged 20 to 80 in a city in northeastern Japan found that eating quickly is significantly associated with an increased risk of overweight (18). Research conducted by O’Connor and colleagues, which included 38,302 adults in the U.S., demonstrates that longer periods of morning fasting are associated with higher obesity rates (19). Existing research has extensively examined the effects of physical activity and diet on BMI. However, most studies tend to focus on each factor in isolation, either considering them separately or treating them as independent variables, thereby overlooking the potential interactive relationship between the two. An online survey conducted in the UK during the COVID-19 social lockdown between April and May 2020 revealed that a higher BMI was associated with lower levels of physical activity and poorer dietary quality (20).

The 19–59 age group is currently the largest demographic in China, and the health status of this group significantly impacts the overall health level of society. Therefore, this study analyzes populations with a BMI < 18.5 kg/m^2^ and BMI ≥ 24 kg/m^2^, examines the relationship between physical activity frequency and BMI among Chinese residents aged 19 to 59, and investigates the role of dietary behavior. The findings aim to provide a basis for future intervention strategies.

## 2 Participants and methods

### 2.1 Study design

This study utilizes a multi-stage sampling method. Initially, the study includes the provincial capitals of 23 provinces, 5 autonomous regions, and 4 directly governed municipalities (Beijing, Tianjin, Shanghai, and Chongqing). Subsequently, a random number table method is used to select between 2 and 6 cities from each non-capital prefecture-level administrative district within the provinces and autonomous regions, resulting in a total of 120 cities. At least one surveyor or one survey team is recruited in each city. Each surveyor is tasked with collecting between 30 and 90 questionnaires, whereas each survey team is tasked with collecting between 100 and 200 questionnaires. Recruitment for this survey excludes the Hong Kong Special Administrative Region, the Macau Special Administrative Region, and Taiwan Province.

The survey was conducted between July 10, 2021, and September 15, 2021. Surveyors utilized the online platform Wenjuanxing^1^ to administer questionnaires both one-on-one and face-to-face to the public in their assigned areas. Respondents completed the questionnaires by clicking on the provided link, gave informed consent during the survey, and the surveyors recorded the questionnaire numbers. If a respondent possesses cognitive abilities but lacks the physical capability to complete the questionnaire, the surveyor will conduct a one-on-one interview and complete the responses on their behalf.

### 2.2 Participants

**Figure 1:**
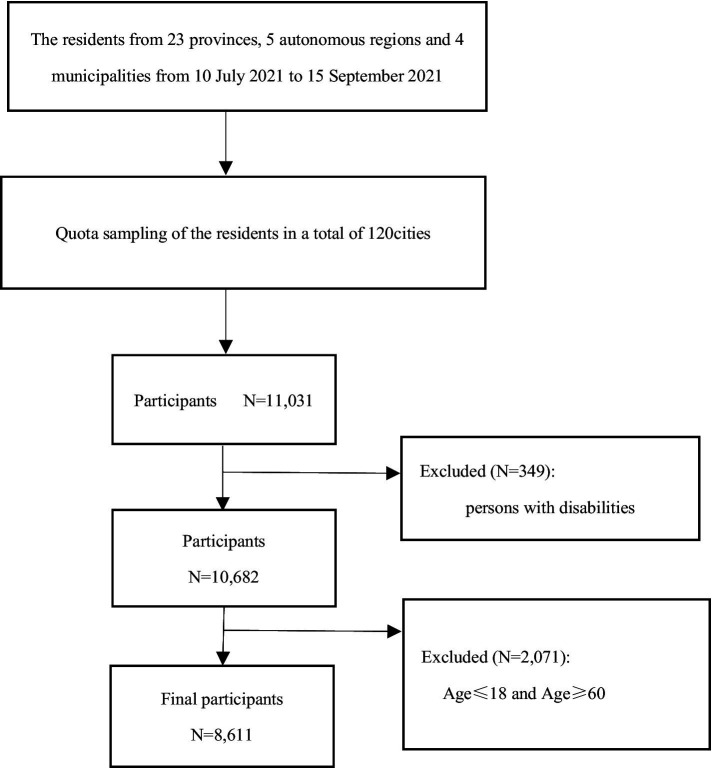
Flow chart of participant enrollment.

The data for this study were sourced from the 2021 China Residents’ Psychological and Behavioral Survey Database. The inclusion and exclusion criteria for the survey conducted in that year are outlined below. Inclusion Criteria: (1) Age ≥ 12 years; (2) Chinese nationality; (3) Resident of China (with no more than 1 month of annual absence); (4) Voluntarily participating in the study and signing the informed consent form; (5) Capable of independently completing the online questionnaire or with the assistance of the surveyor; (6) Understanding the meaning of each item in the questionnaire. Exclusion Criteria: (1) Individuals who are disoriented or have mental abnormalities; (2) Individuals currently participating in similar research studies; (3) Individuals unwilling to cooperate. A total of 11,031 participants from 120 cities across 23 provinces, 5 autonomous regions, and 4 municipalities directly under the central government in China completed the questionnaire. In this study, since the primary focus was on the adult population, data from individuals aged 19 to 59 were specifically selected for analysis. To ensure the representativeness of the sample and the accuracy of the results, individuals who self-reported disabilities, were aged ≤18 years, or were aged ≥60 years were excluded. The final number of respondents included in the study was 8,611, as shown in Figure 1. This study was approved by the ethics review board (JNUKY-2021-018).

### 2.3 Questionnaire

The questionnaire comprises three sections: general demographic information, sport scale, and the short form of the Sakata Eating Behavior Scale (EBS-SF). The general demographic information encompasses gender, age, ethnicity, highest level of education, place of residence, marital status, monthly family income (in RMB), smoking status, alcohol consumption over the past 2 months, as well as self-reported height and weight.

#### 2.3.1 The sport scale

At present, no standardized scale has been specifically developed to assess the frequency of physical activity. The physical activity scale, derived from the first dimension of the Self-Management Scale, is designed to assess the frequency of exercise over a one-week period. It includes six categories: physical training, walking, swimming, cycling, use of fitness equipment, and other aerobic activities (21). The specific details of the scale can be found in Table 1. Each item is rated on a 5-point Likert scale according to the frequency of physical activity, with the following categories: 0 = Not participating, 1 = Less than 30 min per week, 2 = 30 to 59 min per week, 3 = 1 to 3 h per week, and 4 = More than 3 h per week. The scores for all six items are aggregated to yield a total score ranging from 0 to 24, where a higher score signifies a greater frequency of physical activity. The Cronbach’s *α* coefficient for the physical activity scale is 0.814, reflecting a satisfactory level of reliability and affirming that the scale is appropriate for evaluating residents’ physical activity frequency.

**Table 1:** Respondents’ physical activity level in the past week (*N* = 8,611).

	None, *N* (%)	<30 minutes/week, *N* (%)	30–59 minutes/week, *N* (%)	1–3 hours/week, *N* (%)	>3 hours/week, *N* (%)
Fitness exercises (such as circuit training, weightlifting, etc.)	3100 (36)	2092 (24.3)	1345 (15.6)	1308 (15.2)	766 (8.9)
Walking	871 (10.1)	1723 (20.0)	2025 (23.5)	2025 (23.5)	1967 (22.8)
Swimming	5980 (69.4)	1052 (12.2)	683 (7.9)	652 (7.6)	244 (2.8)
Cycling	4222 (49.0)	1545 (17.9)	1242 (14.4)	1023 (11.9)	579 (6.7)
Exercising with equipment (such as a treadmill, trampoline, etc.)	5153 (59.8)	1322 (15.4)	918 (10.7)	798 (9.3)	420 (4.9)
Other aerobic exercises (such as running, playing table tennis, etc.)	3115 (36.2)	1821 (21.1)	1555 (18.1)	1262 (14.7)	858 (10.0)

#### 2.3.2 The Sakata Eating Behavior Scale short form

This study employed a modified version of the dietary behavior scale to evaluate residents’ dietary behaviors. The Sakata Eating Behavior Scale Short Form (EBS-SF) is a reliable and effective instrument designed to assess abnormal eating behaviors associated with obesity (22). The EBS-SF employs a 4-point Likert scale for scoring, which reflects residents’ level of agreement with the statements (1 = Strongly Disagree, 2 = Disagree, 3 = Agree, 4 = Strongly Agree). The total score ranges from 7 to 28, based on seven items, with higher scores indicating poorer eating behaviors. In this study, the Cronbach’s *α* coefficient for the EBS-SF was 0.873, demonstrating good internal consistency.

### 2.4 Statistical analysis

The data were statistically described and analyzed using SPSS version 26.0 and R version 4.2.0 software. For non-normally distributed quantitative data, medians and interquartile ranges (IQRs) were used for statistical descriptions. Categorical data were presented as frequencies and percentages. The chi-square test was employed for categorical data, whereas non-parametric tests were applied to quantitative data that did not follow a normal distribution or to categorical data. To investigate the factors influencing body mass index (BMI), this study utilized multiple logistic regression analysis, with individuals having a BMI between 18.5 and 23.9 as the reference group. Initially, *Z*-score standardization was performed on the EBS-SF to eliminate variations and improve data comparability and analytical precision. Subsequently, the mediation package in RStudio was employed to examine the mediating effect of dietary behavior on the relationship between exercise frequency and body mass index (BMI). Concurrently, the glmnet package was utilized for regularized regression analysis to optimize the model and manage high-dimensional data, thereby ensuring the reliability of the results. All tests were conducted with a significance level of *α* = 0.05.

## 3 Results

### 3.1 Basic information

In this survey, a total of 8,611 individuals were sampled: 1,066 (12.38%) had a BMI < 18.5 kg/m^2^, 5,354 (62.18%) had a BMI between 18.5 and 23.9, and 2,191 (25.44%) had a BMI ≥ 24 kg/m^2^. The overall score on the physical activity scale was 6.00 (4.00, 11.00). For individuals with a BMI < 18.5 kg/m^2^, the score was 6.00 (3.00, 11.00). For individuals with a BMI between 18.5 and 23.9, the score was 6.00 (4.00, 11.00). For individuals with a BMI ≥ 24 kg/m^2^, the score was 6.00 (3.00, 11.00).

Over the past week, swimming, using exercise equipment, and cycling were the activities with the lowest participation rates, whereas walking was the most frequently engaged activity. For further details, refer to Table 1. The overall score on the dietary behavior scale was 17.00 (14.00, 20.00). For individuals with a BMI < 18.5 kg/m^2^, the score was 17.00 (14.00, 20.00). For individuals with a BMI between 18.5 and 23.9, the score was 16.00 (14.00, 20.00). For individuals with a BMI ≥ 24 kg/m^2^, the score was 17.00 (15.00, 21.00). The three most frequently endorsed abnormal eating behaviors among participants, as shown in Table 2, are “eating quickly,” “believing oneself to be more prone to weight gain than others,” and “eating when others around me are eating. For further details, refer to Table 2.

**Table 2:** Summary of scores on the short version of the eating behavior scale (*N* = 8,611).

	Strongly disagree	Moderately disagree	Moderately agree	Strongly agree
No fixed mealtimes.	1790 (20.79)	2775 (32.23)	3441 (39.96)	605 (7.03)
Only when very full am I satisfied.	1712 (19.88)	3501 (40.66)	2729 (31.69)	669 (7.77)
Fast eating speed.	1231 (14.30)	2641 (30.67)	3776 (43.85)	963 (11.18)
I think I am more prone to gaining weight than others.	1374 (15.96)	2471 (28.70)	3503 (40.68)	1263 (14.67)
I like fatty foods.	1501 (17.43)	3005 (34.90)	3411 (39.61)	694 (8.06)
If people around me are eating, I eat too.	1252 (14.54)	3306 (38.39)	3402 (39.51)	651 (7.56)
When I buy food, I am only satisfied if I buy more than I need.	1865 (21.66)	3456 (40.13)	2679 (31.11)	611 (7.10)

### 3.2 Comparison of BMI among different demographic groups

The results indicate that BMI variations among different genders, ages, ethnicities, educational levels, marital statuses, smoking statuses, monthly household incomes, occupational statuses, alcohol consumption in the past 12 months, exercise scale scores, and dietary behavior scale scores were statistically significant (*p* < 0.05), as presented in Table 3.

**Table 3:** Comparison of BMI differences among respondents with different characteristics (*N* = 8,611).

Variables	Total	Body Mass Index			*p-*value
		< 18.5	18.5–23.9	≥ 24	
Subjects, *n* (%)	8611	1066 (12.38)	5354 (62.18)	2191 (25.44)	
Gender, *n* (%)					<0.001
Man	3895 (45.23)	290 (27.20)	2297 (42.90)	1308 (59.70)	
Woman	4716 (54.77)	776 (72.80)	3057 (57.10)	883 (40.30)	
Age, *n* (%)					<0.001
19–35	4455 (51.74)	808 (75.80)	2881 (53.81)	766 (34.96)	
36–45	2021 (23.47)	146 (13.70)	1263 (23.59)	612 (27.93)	
46–59	2135 (24.79)	112 (10.51)	1210 (22.60)	813 (37.11)	
Smoking status, *n* (%)					<0.001
Never smoked	6922 (80.39)	941 (88.27)	4455 (83.21)	1526 (69.65)	
smoking	1175 (13.65)	92 (8.63)	596 (11.13)	487 (22.23)	
Abandon smoking	514 (5.97)	33 (3.10)	303 (5.66)	178 (8.12)	
Drinking status, *n* (%)					<0.001
Never drank	3728 (43.30)	394 (37.00)	2161 (40.40)	1173 (53.50)	
Drinking	4883 (56.70)	672 (63.00)	3193 (59.60)	1017 (46.50)	
Education level, *n* (%)					<0.001
Primary education and below	465 (5.4)	45 (4.22)	266 (4.97)	154 (7.03)	
Secondary education	2322 (26.97)	177 (16.60)	1384 (25.85)	761 (34.73)	
Higher education	5824 (67.63)	844 (79.17)	3704 (69.18)	1276 (58.24)	
Nation, *n* (%)					0.037
Han	8129 (94.40)	989 (92.78)	5060 (94.51)	2080 (94.93)	
Minority nationality	482 (5.60)	77 (7.22)	294 (5.49)	111 (5.07)	
Marital status, *n* (%)					<0.001
Married	5205 (60.44)	396 (37.15)	3170 (59.20)	1639 (74.80)	
Unmarried	3192 (37.07)	647 (60.69)	2060 (38.48)	485 (22.14)	
Other	214 (2.49)	23 (2.16)	124 (2.32)	67 (3.06)	
Per capita monthly income, *n* (%)					0.014
<4500	4164 (48.36)	536 (50.28)	2568 (47.96)	1060 (48.38)	
4500-9000	3025 (35.13)	335 (31.43)	1941 (36.25)	749 (34.19)	
>9000	1422 (16.51)	195 (18.29)	845 (15.78)	382 (17.43)	
Occupational status, *n* (%)					<0.001
Student	2199 (25.54)	466 (43.71)	1422 (26.56)	311 (14.19)	
On-the-job	4521 (52.50)	430 (40.34)	2803 (52.35)	1288 (58.79)	
Other	1891 (21.96)	170 (15.95)	1129 (21.09)	592 (27.02)	
Place of residence, *n* (%)					0.342
Rural	2146 (24.92)	285 (26.75)	1322 (24.69)	539 (24.60)	
Urban	6465 (75.08)	781 (73.26)	4032 (75.31)	1652 (75.40)	
Physical activity level	6.00 (4.00, 11.00)	6.00 (3.00, 11.00)	6.00 (4.00, 11.00)	6.00 (3.00, 11.00)	<0.001
EBS-SF	17.00 (14.00, 20.00)	17.00 (14.00, 20.00)	16.00 (14.00, 20.00)	17.00 (15.00, 21.00)	<0.001

### 3.3 Results of the multivariate logistic regression analysis of BMI

BMI was used as the dependent variable, with normal BMI (18.5 kg/m^2^ < BMI < 24 kg/m^2^) serving as the reference group. Variables that were statistically significant in the univariate analysis were subsequently included as independent variables in the multivariate logistic regression analysis. Detailed results are provided in Table 4. Compared to the normal BMI group, females are more likely to have a BMI < 18.5 kg/m^2^ [AOR: 2.209 (1.862–2.621)], while males are more likely to have a BMI ≥ 24 kg/m^2^ [AOR: 0.572 (0.505–0.647)]. Age is both a protective factor for BMI < 18.5 kg/m^2^ [46–59: AOR: 0.469 (0.364–0.604), 36–45: AOR: 0.570 (0.453–0.717)] and a risk factor for BMI ≥ 24 kg/m^2^ [46–59: AOR: 1.920 (1.642–2.244), 36–45: AOR: 1.449 (1.239–1.695)]. The risk of having a BMI < 18.5 kg/m^2^is 1.634 times higher in the unmarried group compared to the married group [AOR: 1.634 (1.314–2.032)]. Being unmarried is associated with a lower likelihood of having a BMI ≥ 24 kg/m^2^ [AOR: 0.662 (0.546–0.802)]. Individuals with a household monthly income >9,000 yuan are less likely to have a BMI < 18.5 kg/m^2^ compared to those with an income ≤4,500 yuan [AOR: 1.290 (1.064–1.563)]. Individuals with higher education are less likely to have a BMI < 18.5 kg/m^2^ compared to those with only primary education or less [AOR: 0.684 (0.475–0.984)]. Smoking is a risk factor for both a BMI < 18.5 kg/m^2^ [AOR: 1.431 (1.097–1.868)] and a BMI ≥ 24 kg/m^2^ [AOR: 1.253 (1.068–1.469)]. Additionally, both alcohol consumption [AOR: 1.326 (1.180–1.490)] and abnormal eating behaviors [AOR: 1.078 (1.065–1.091)] are risk factors for a BMI ≥ 24 kg/m^2^. Exercise is a protective factor for a BMI ≥ 24 kg/m^2^ [AOR: 0.972 (0.963–0.982)].

**Table 4:** Results of multivariate logistic regression associated with BMI (*N* = 8,611).

Variables (Reference)	BMI<18.5		BMI≥24	
	A_OR_ (95%CI)	*p*-value	A_OR_ (95%CI)	*p*-value
Gender (Male)	2.209 (1.862–2.621)	<0.001	0.572 (0.505–0.647)	<0.001
Age (19–35)				
36-45	0.469 (0.364–0.604)	<0.001	1.920 (1.642–2.244)	<0.001
46-59	0.570 (0.453–0.717)	<0.001	1.449 (1.239–1.695)	<0.001
Nation (Han)	1.247 (0.954–1.630)	0.106	0.909 (0.719–1.151)	0.430
Marital status (Married)				
Other	1.441 (0.906-2.291)	0.123	1.032 (0.754-1.412)	0.845
Unmarried	1.634 (1.314–2.032)	<0.001	0.662 (0.546–0.802)	<0.001
Per capita monthly income (≤4500)				
>9000	1.290 (1.064–1.563)	0.010	1.107 (0.950–1.291)	0.192
4501–9000	0.952 (0.814–1.114)	0.539	0.925 (0.820–1.043)	0.203
Education level (Secondary education)				
Primary education and below	0.764 (0.531–1.101)	0.149	0.954 (0.752–1.210)	0.698
Higher education	0.684 (0.475–0.984)	0.041	1.042 (0.829–1.310)	0.723
Smoking status (Never smoked)				
Abandon smoking	1.102 (0.747–1.627)	0.623	0.882 (0.712–1.092)	0.250
smoking	1.431 (1.097–1.868)	0.008	1.253 (1.068–1.469)	0.006
Drinking status (Never drank)				
Drinking	0.995 (0.825–1.328)	0.946	1.326 (1.180–1.490)	<0.001
Occupational status (on-the-job)				
Other	1.078 (0.873-1.332)	0.486	1.038 (0.904, 1.191)	0.600
Student	1.157 (0.948–1.413)	0.151	0.871 (0.710–1.069)	0.188
EBS-SF	0.995 (0.980–1.010)	0.532	1.078 (1.065–1.091)	<0.001
Physical activity level	0.995 (0.–1.008)	0.473	0.972 (0.963–0.982)	<0.001

### 3.4 Results of mediation effects analysis

**Figure 2:**
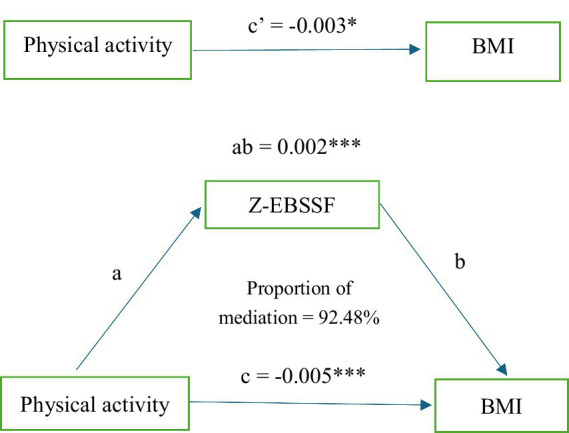
Mediated model of Z-EBSSF. Path a is a regression analysis between exposure and mediator. Path b is a regression analysis between mediator and outcome. Path c is a regression analysis between exposure and outcome. Path c’ is a regression analysis between exposure and outcome. The indirect effect (ab) estimate is the amount of mediator contribution to the relationship between exposure and outcome. * *p*-value <0.05. * * *p*-value <0.001.

The KHB method was employed to examine the mediating role of dietary behavior in the relationship between physical activity and abnormal BMI. The results are illustrated in Figure 2. Our analysis revealed that dietary behavior mediates the relationship between physical activity and abnormal BMI. The total effect of physical activity on abnormal BMI was −0.003, with a direct effect of −0.005. The mediating effect of dietary behavior in the relationship between physical activity and abnormal BMI was 0.002, constituting 92.48% of the total effect. For further details, please refer to Table 5.

**Table 5:** Mediating effects of Z-EBSSF in physical activity level and BMI.

Variables	*β* (95% CI)	SE	Proportion	*p*
Total effect	−0.0026 (−0.0049, −0.0002)	0.001	-	0.037
Direct effect	−0.0050 (−0.0073, −0.0027)	0.001	-	<0.001
Mediation effect	0.0024 (0.0019, 0.0030)	<0.001	92.48%	<0.001

Z-EBS-SF, The Short-Form of the Eating Behavior Scale; BMI, body mass index.

## 4 Discussion

In this survey, the mean BMI was 22.04 ± 3.19 kg/m^2^, with 12.38% of the population classified as underweight (<18.5 kg/m^2^) and 25.44% classified as overweight (≥24 kg/m^2^). Another survey involving approximately 15.77 million Chinese participants aged 18 years and older indicates that the mean BMI in China is 24.1 kg/m^2^, with an overweight rate of 34.8% and an obesity rate of 14.1% (23). The results of this study are higher than those of our research, which may be attributable to variations in sample selection or research methodology.

In this study, we observed that individuals aged 35–59 years exhibited a higher risk of being overweight compared to those aged 19–35 years. This trend may be attributable to a decline in metabolic rate. Additionally, there are significant differences in BMI outcomes based on gender. Research indicates that women are more likely to have a lower BMI compared to men, which is associated with physiological characteristics and societal expectations (24, 25). A study involving 18,512 university students from 22 countries found that even when women’s BMI has not yet reached the medical threshold for obesity or overweight, they may still perceive the need to lose weight. In contrast, men are less likely to recognize that they are overweight and are less inclined to attempt weight loss (25). The influence of marital status on BMI is also evident (26). Married individuals are at a higher risk of adverse BMI outcomes compared to unmarried individuals, which may be related to lifestyle changes associated with marriage. For instance, the “happy weight gain” phenomenon suggests that satisfaction in intimate relationships may contribute to weight gain (27). Conversely, this study found that the BMI of unmarried individuals is lower, which may be attributed to their more frequent social activities, focus on appearance, or healthier eating habits. The influence of education and income levels on BMI should not be overlooked. Large-scale surveys in South Korea indicate that low-income groups are more likely to experience severe underweight issues, while high-income groups exhibit relative stability (28). Similarly, research in the U.S. has found that higher education levels are associated with lower BMI, likely due to increased health awareness and improved dietary habits associated with higher education (29). Furthermore, this study found that smokers and drinkers are more likely to experience weight abnormalities (such as being underweight or overweight) compared to non-smokers and non-drinkers, consistent with previous research (30).

With the rapid economic development, dietary patterns among Chinese residents have undergone significant changes. There has been a sharp increase in the consumption of high-sugar and high-calorie foods; concurrently, rates of obesity and overweight have risen significantly (31). These phenomena further underscore the adverse impact of poor dietary habits on individuals’ Body Mass Index (BMI). Currently, an increasing number of individuals are managing their BMI through dietary control (32). A study conducted in Japan indicated that individuals with more intense dieting behaviors exhibit smaller discrepancies between their actual weight and their ideal weight, BMI, and body shape (33). Diet control is achieved by adjusting an individual’s intake of dietary components, whereas behavioral change focuses on achieving this through nutritional education and the establishment of healthy eating habits. In this study, abnormal dietary behaviors are identified as risk factors for an overweight BMI. Conversely, a healthy lifestyle aids individuals in maintaining a normal BMI. Physical activity is regarded as a crucial factor for promoting long-term weight loss and preventing weight regain (17, 34, 35). Although methods such as behavior modification, medication, and surgery can reduce obesity, increasing physical activity is often a more preferable option when considering socioeconomic factors and potential side effects (36). This study finds that individuals with higher exercise frequencies are less likely to exhibit abnormal BMI, which is consistent with previous research (37). Additionally, physical activity is a factor that promotes body development, thereby aiding individuals in maintaining a normal BMI (38).

It is noteworthy that, in the mediation effect analysis, abnormal dietary behaviors were found to obscure the protective effect of exercise on abnormal BMI. Increasing the frequency of exercise helps mitigate the risk of abnormal BMI. However, the presence of abnormal dietary behaviors significantly diminished this protective effect. Furthermore, this study revealed that abnormal dietary behaviors obscured 92.48% of the protective effect of exercise on abnormal BMI. This masking effect indicates that abnormal dietary behaviors could be a crucial intervention point or influencing factor, playing a pivotal role in the relationship between exercise and abnormal BMI. Abnormal dietary behaviors, such as rapid eating, can result in excessive food intake because the brain requires time to process satiety signals (39). Numerous studies indicate that individuals who eat rapidly are more prone to consuming excess calories, which can lead to weight gain, whereas eating slowly may enhance satiety and aid in controlling food intake (40, 41). Abnormal eating behaviors can impair digestive efficiency, leading to symptoms such as indigestion or bloating, which may affect comfort and performance during exercise (42, 43). Therefore, future research should further investigate effective interventions for abnormal eating behaviors to optimize exercise outcomes and mitigate the risk of abnormal BMI. Additionally, a comprehensive intervention customized to individual dietary habits and exercise patterns may be essential for enhancing overall health. It is crucial to recognize that individual dietary habits are shaped by regional cultural influences. Therefore, it is essential to take the local dietary context into account when designing and implementing intervention strategies.

Although this is a large-scale, national cross-sectional study, it has several limitations. Firstly, because the national sample data is cross-sectional, it is not possible to analyze temporal changes in correlations (44). Secondly, as weight and height are self-reported, this could result in an underestimation of BMI. Some studies have indicated that the discrepancy between self-reported and measured weight is relatively small among overweight individuals, this bias may still influence the accuracy of the results (45). Additionally, the sample in this study predominantly comprised adults aged 19 to 59, which may limit the external validity of the findings when applied to child and older adult populations. Future research should further explore the patterns of BMI variation across different age groups and the determinants driving these changes. Furthermore, this study did not adequately account for cultural differences in diet, which may impact the applicability of the findings across different regions and populations.

## 5 Conclusion

This study indicates that 37.82% of Chinese adults aged 19–59 have an abnormal BMI, with a higher proportion of individuals exhibiting a BMI ≥ 24 kg/m^2^. Although increased physical activity has a positive effect on BMI control, poor dietary behaviors significantly undermine the effectiveness of exercise. Therefore, it is essential to raise awareness of abnormal BMI and implement effective intervention strategies. The study also found that exercise alone is insufficient to significantly improve BMI; combining physical activity with dietary improvements is a more effective approach. Future research should further investigate the differential responses to dietary and exercise interventions across various populations (e.g., by age, gender, region, etc.) in order to develop more targeted health management strategies.

## Data Availability

The raw data supporting the conclusions of this article will be made available by the authors, without undue reservation.

## Footnotes

^1^ https://www.wjx.cn/
